# Aizoon extract as an eco-friendly corrosion inhibitor for stainless steel 430 in HCl solution

**DOI:** 10.1039/d2ra05795f

**Published:** 2022-10-28

**Authors:** Abd El-Aziz S. Fouda, Ameena M. Al-Bonayan, Ahmed F. Molouk, M. Eissa

**Affiliations:** Chemistry Department, Faculty of Science, Mansoura University Mansoura-35516 Egypt asfouda@hotmail.com; Chemistry Department, Faculty of Science, Umm Al-Qura University Makkah Kingdom of Saudi Arabia; Higher Institute of Engineering & Technology, KMA Alex Egypt; Huraymila College, Chemistry Department, Al Imam Mohammad Ibn Saud Islamic University (IMSIU) Kingdom of Saudi Arabia

## Abstract

Aizoon extract is used as an eco-friendly anti-corrosive material for stainless steel 430 (SS430) in a 2 M hydrochloric acid solution. Many strategies were utilized to estimate the mitigation efficacy such as mass reduction (MR), electrochemical impedance spectroscopy (EIS), and potentiodynamic polarization (PDP). The inhibition percentage (%*I*) increases by increasing the concentration of Aizoon and reaches 95.8% at 300 ppm and 298 K, while it lowers by raising the temperature, reaching 85.6% at 318 K. Tafel curves demonstrated that Aizoon extract is a mixed type inhibitor with an excellent ability to inhibit the cathodic reaction. Adsorption of the Aizoon extract on an SS430 surface is regulated by the Langmuir adsorption model. The value is 
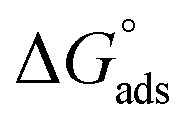
 is −20.9 kJ mol^−1^ at 298 K indicating that the adsorption is of mixed type affecting both cathodic and anodic reactions. Thermodynamic factors for adsorption and activation processes were estimated and discussed. The adsorption of Aizoon extract on the SS430 surface was tested utilizing Fourier transform infrared spectroscopy (FTIR) and scanning electron microscope (SEM) techniques. The Nyquist curves confirmed that Aizoon extract prohibits the disintegration of SS430 in an acid medium without changing the dissolution reaction mechanism. The theoretical calculations showed that Aizoon extract is considered as an excellent corrosion inhibitor. The experimental data were supported by theoretical evaluations.

## Abstract 2

Aizoon extract is used as an eco-friendly anti-corrosive material for stainless steel 430 (SS430) in 2 M hydrochloric acid solution.

## Graphical abstract

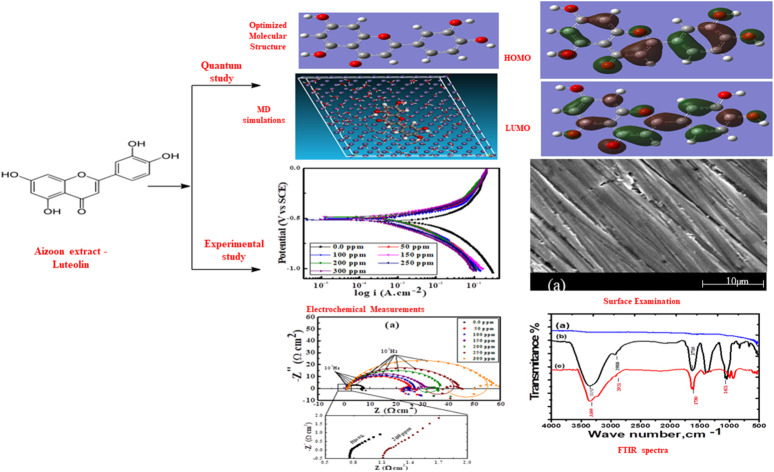

## Introduction

Because of its vast range of uses in industry, such as automobile accessories, culinary utensils, cleaning equipment components, and industrial pipes, global stainless steel (SS) production has increased rapidly in recent decades. Furthermore, the tanks used to transport nitric acid are frequently constructed of SS430. Because of the development of a thin passive coating of chromium ox-hydroxide that protects the alloy, stainless steel has high corrosion resistance.^1^ However, in an acidic medium such as HCl, the passive film formed over the SS surface disappears as chromium reacts with chloride producing chromium chloride. The chromium will be gradually lost from SS leaving just iron.^2^ Therefore, using corrosion inhibitors is a very beneficial strategy for protecting SS against corrosion as these compounds are adsorbed on SS active sites and in turn protect it from a corrosive medium. Organic compounds are the most dominant compounds used in industry. Often, the chemical structure of efficient compounds should contain donor atoms such as oxygen, sulfur, and nitrogen and/or double and triple bonds which assists the absorption of these organic compounds on the metal surface through bonding the electron pair of the donor atoms with the active sites on metal surface. Consequently, the corrosive attack in an acidic medium will be retarded.^3–5^ Despite the high efficiency of organic compounds, they have a lot of drawbacks due to the environmental problems associated with them even if they are added in small concentration. The majorities of the chemicals examined are hazardous and pose a serious threat to the environment. Attention has shifted to naturally occurring substances because they are environmentally friendly. Several authors have written about the use of natural compounds as corrosion inhibitors. Plant extracts' corrosion inhibitory activity could be attributed to the presence of heterocyclic constituents such as alkaloids, flavonoids, and other flavonoids; however, the presence of tannins, cellulose, and polycyclic compounds enhances the formation of a film over the metal surface, which aids corrosion protection. Plant extracts have several advantages over traditional hazardous and non-environmental corrosion inhibitors, such as significant efficiency, widespread commercial accessibility, non-hazardous characteristics, cost-effectiveness, and reduced hazards to the soil, aquatic and terrestrial life.^6^ Numerous researches were carried out using extracts from plants as successful anti-corrosion for iron and steel in acidic environments. Qiang *et al.* investigated the inhibition influence of extract from ginkgo leaves on X70 steel in 1 M HCl by conducting electrochemical measurements.^7^ The inhibition efficiency exceeded 90% by adding 200 ppm of the extract at all tested temperatures demonstrating an excellent inhibition capacity. In other research, the inhibition characteristics were investigated for the extract obtained from *Dendrocalamus brandisii* leaves to protect cold rolled steel in trichloroacetic acid by measuring the loss in weight supported by electrochemical tests. The results showed that the extract performs well with a maximum inhibition percentage of more than 95%.^8^ A study was done by Suprapto *et al.* on the performance of Acera Alba extract as anti-corrosion for SS304 in HCl. The polarization study revealed that the extract acts as a mixed extract giving inhibition up to 90% and the adsorption on SS304 obeyed Frumkin isotherm.^9^ Moreover, Gapsari *et al.* confirmed the effectiveness of an extract from bee wax propolis on the SS304 in 0.5 M sulfuric acid. The results showed that the extract with a concentration of 2000 ppm retarded the corrosion rate and the inhibition percentage reached 97.29%.^10^ In 0.5 M H_2_SO_4_ media, Z. Lin *et al.*^11^ investigated Locust Bean Gum (LBG) as a novel and environmentally benign corrosion inhibitor for Q235 steel, inhibition efficiency was 89.8% with the concentration 5 mM at 298 H. Tian *et al*.^12^ prepared Algae extract and used it as a green corrosion inhibitor for Q235 steel in chloride ion solutions the inhibition efficiency reached 82.1% at 300 ppm and 30 °C. For the dissolution of metals in acid solutions, numerous plant extracts have been used effectively and efficiently such as: *Cryptocarya nigra* extracts,^13^ multi-phytoconstituents from *Dioscorea septemloba*,^14^*Euphorbia heterophylla* L. extract,^15^ physically modified starch,^16^ methanolic myrrh extract,^17^*Hemerocallis fulva*,^18^*Rosa canina* fruit extract,^19^ aqueous Chinese gooseberry fruit shell extract,^20^*Ficus hispida* leaves,^21^ radish leaf extract.^22^ Therefore, plant extracts are promising agents for protecting metals and alloys from corrosion. The data gained from the various studies showed that plant extracts could serve as effective inhibitors for corrosion. The plant extract, Aizoon, is a genus of flowering plants in the ice plant family of Aizoaceae it is originating in the Mediterranean region, and widely cultivated in North Africa from the Atlantic Islands (Canaries) and Cape Verde eastward *via* northern Africa to Egypt and Sudan. On the other hand, there is a shortage stage in the research that study the protection of SS430 from corrosion in an acidic medium compared to other SS grades. In this work the Aizoon extract was selected to be investigated because of its constituent compounds are promising as corrosion inhibitors^23^ besides its availability in several regions and ease to obtain the extract.

The goal of this research is to examine how Aizoon extract reduces SS430 disintegration in 2 M HCl pickling solution using mass reduction (MR), potentiodynamic polarization (PDP), and electrochemical impedance spectroscopy (EIS) tests. The adsorption performance of eco-friendly environment Aizoon extract on SS430 was investigated. The study also comprises the impact of temperature on inhibition efficiency. In addition, the surface of SS430 samples devoid of and with Aizoon extract was examined.

## Experimental methods

### Chemical composition and extraction of Aizoon plant

Eleven compounds were identified for Aizoon extract hydroxybenzoic acid (1), gallic acid (2), protocatechuic acid (3), vallinic acid (4), thymine (5), caffeic acid (6), 5,7-dihydroxy chromone (7), pyrogallol (8), quercetin (9), kaempferol (10), and luteolin (11).^24^
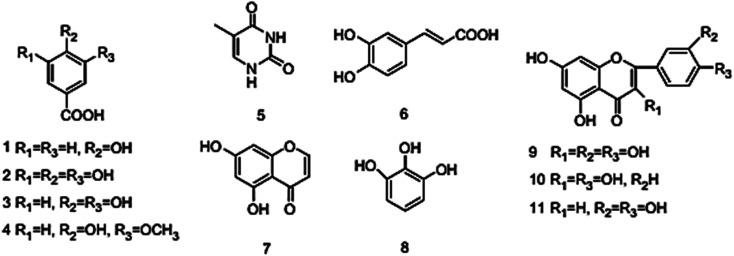


Plant used in this work was collected from Sadat city desert, Egypt. Leaves of Aizoon were pre-washed and then dried in air at room temperature away from direct light for several days. The dried leaves were coarsely crushed in a cylindrical crusher and then sieved to obtain a homogeneous powder. The extract was obtained by soaking 200 g of the leaves powder in 200 mL methanol 70% (ADWIC, Nasr Pharma, Egypt) for 48 h. The powder was removed by filtration to get a clear filtrate. Subsequently, the filtrate was vaporized under a vacuum. The residue after evaporation was collected and stored below 273 K in a glass flask stoppered with a screw plastic lid. It was confirmed that the Aizoon extract consists of eleven compounds identified as: 5,7-dihydroxy chromone, pyrogallol, kaempferol, protocatechuic acid, vallinic acid, thymine, caffeic acid, quercetin, *p*-hydroxybenzoic acid, gallic acid, and luteolin.^25^

### Chemical composition of SS430 and its pretreatment

#### A ferritic category of steels includes (wt%)

Carbon was 0.12 percent, manganese was 1.0, phosphorus was 0.045, chromium was 16 sulfur was 0.03, silicon was 1.0, nickel was 0.5, and iron made up the remainder. Before testing, SS430 samples were cleaned with double-distilled water, degreased with acetone (ADWIC, Nasr Pharma, Egypt), and polished with various grades of silicon carbide. The coupons were ultimately dried out at room temperature and weighed using an analytical balance.

### Preparation of pickling solution

The corrosive media for SS430 specimens is 2 M HCl. It was made using double-distilled water and analytical grade condensed 37% HCl.

### Mass reduction (MR) measurements

MR tests were fulfilled based on the American Society for Testing and Materials. All SS430 coupons used in MR experiments were mechanically cut into a tetragonal shape of 20 × 20 × 2 mm (two faces) dimensions with an effective surface area of 800 mm^2^. The concentration range of Aizoon extract in test solutions was selected to be 0–300 ppm. The weighted SS430 coupons were immersed for 180 minutes in 100 mL of 2 M hydrochloric acid without and with varying amounts of Aizoon extract. The coupons were removed from a solution at various intervals of time (30 min), rinsed, dried, and then accurately weighed. Each experiment was performed at least three times to verify repeatability.

### Electrochemical measurements

The behavior of Aizoon extract as an anti-corrosion for SS430 in 2 M HCl was further examined by PDP and EIS tests. All electrochemical experiments were performed at 298 K using a three-electrode setup consisting of a saturated calomel electrode (SCE) as the reference, platinum foil as an auxiliary, and SS430 as a working (WE) electrodes in 100 mL glass cell. The WE was prepared as follows. One side of the SS430 sheet (10 × 10 × 2 mm) was connected to a copper wire for electric connection. Subsequently, the copper wire attached to the SS430 sheet was inserted into a glass tube and then fixed by epoxy resin to make a 1 cm^2^ area of SS430 exposed to the solutions. Before measurements the samples were pretreated as mentioned before and the potential of the electrode was stabilized for 30 min to reach a steady state. For the PDP test, the potential was scanned from −0.5 to 0.5 V against open circuit potential (OCP) with a scan rate of 0.2 mVs^−1^. AC impedance tests were carried out in a frequency range from 0.1 to 10^−5^ Hz with an amplitude of 10 mV peak to peak under open circuit conditions. The experimental data collected from EIS measurements were analyzed based on the equivalent circuit. However, polarization resistance *R*_p_, were estimated from the distance between the high-and low-frequency intercepts with the abscissas for the impedance spectra. All electrochemical tests were performed using Gamry instrument Potentiostat/Galvanostat/ZRA (PCI4-G750). The utilized software is DC105 for PDP, EIS300 for EIS, and the data obtained were analyzed using Echem analyst v 5.5 software.

### Surface characterization

SS430 samples used for surface analysis were submerged at 298 K for 3 h in extract-free acid and acid containing 300 ppm of Aizoon extract. After that, the samples were removed cleaned with double distilled water many times to remove any residue and dried. The surface morphology of SS430 samples was characterized using a scanning electron microscope (SEM) (JOEL 840, Japan). The adsorption of Aizoon extract on SS430 surface was investigated by Fourier transform infrared spectroscopy (FTIR) Thermo Fisher Nicolet IS10, USA in the spectral range of 400–4000 cm^−1”^. The FTIR spectra were recorded for SS430 surface before and after 3 h immersion in acid–Aizoon mixture (300 ppm Aizoon in 2 M HCl) and then compared to the spectra of Aizoon extract. Before the FTIR measures the SS430 sample immersed in acid–Aizoon mixture was rinsed several times with ethanol and water to remove any Aizoon residue from the SS430 surface.

### Theoretical calculation details

Materials Studio 2017 software was used to determine the quantum chemical characteristics of the Locust Bean Gum (LBG) molecule using the Dmol3module. Structure optimization was chosen as the calculation task. The amount of energy was 4 × 10^5^ ha. The maximum force measured was 0.05 Hz. DNP and 4.4 were the basis sets and files, respectively. The Force field was chosen to be COMPASS. Materials Studio's cite module was used to do Molecular Dynamics Simulations (MDS) of Aizoon molecule adsorption on the surface of Fe (110). The vacuum layer of 30 A was chosen to ensure that the repeated slabs were decoupled. All Fe atoms' positions are fixed. As research targets, 400 water molecules and one Aizoon molecule were chosen. The quality was average. NVT was the ensemble. The simulation took 500 ps in total.

## Result and discussion

### Mass reduction (MR) tests

The MR values, corrosion rate (CR), inhibition percent (*I*%), and surface coverage (*θ*) for SS430/2 M HCl system in the presence of different Aizoon concentrations (0–300 ppm) at 298–318 K obtained from MR experiments after 3 h immersion are summarized in Table 1 and calculated through the following equations.

**Fig. 1:**
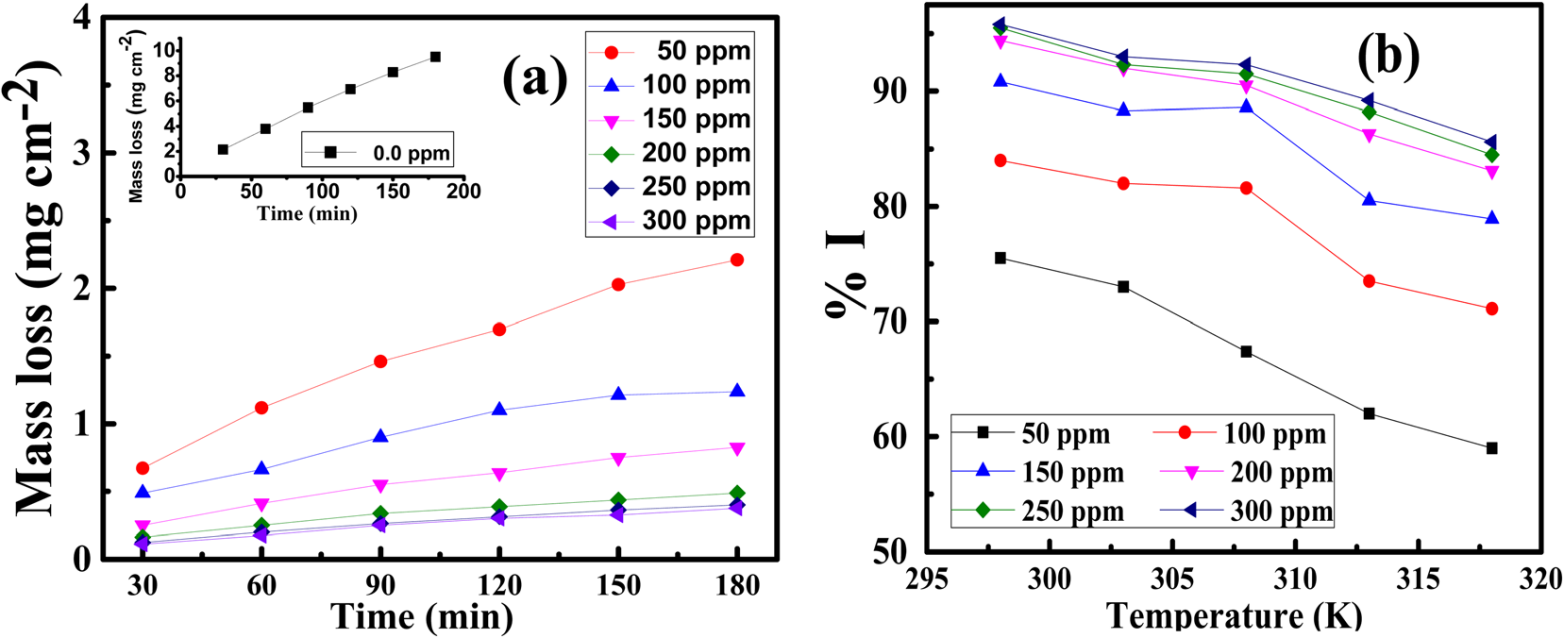
(a) MR–time curves at 298 K and (b) inhibition percent as a function of temperature for SS430/2 M HCl in the presence of various Aizoon concentrations.

1 MR = *m*_2_ − *m*_1_

where *m*_1_ and *m*_2_ are the weights of the SS430 sheet prior to and after submersion in the test solution, respectively.2
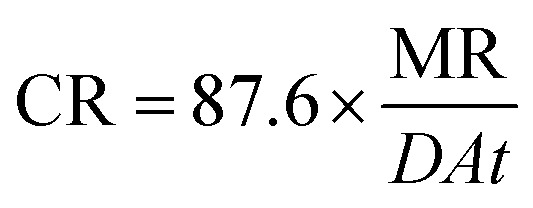
where MR is the loss in mass, *D* is the alloy density, *t* is the submersion time and *A* is the active area exposed to the test solution.3
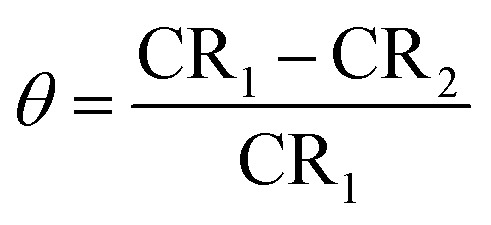
4
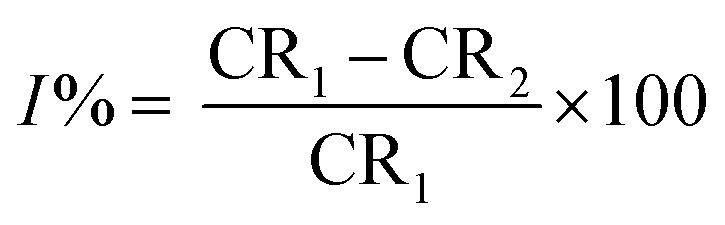
where CR_1_ and CR_2_ are the corrosion rates of SS430 without and with Aizoon extract, respectively. Fig. 1(a) shows the mass reduction–time curves for SS430/2 M HCl for different Aizoon concentrations at 298 K. Similar curves were done for other temperatures (not shown). It can be observed that the addition of Aizoon extract is accompanied by decreases in the mass reduction and corrosion rate, on the other hand, the surface coverage and the inhibition percent increased reaching 95.8% of maximum inhibition at 300 ppm Aizoon concentration. This confirms the inhibition characteristic of Aizoon extract for protecting SS430 corrosion in an acidic medium. Raising the temperature of the test leads to a significant increase in mass reduction and corrosion rate of SS430 for Aizoon concentrations compared to those at 298 K (see Table 2). Therefore, the %*I* of the extract decreases with raising temperature as seen in Fig. 1(b) which summarizes the inhibition efficiency as a function of temperature for all Aizoon concentrations. This can be explained by the decrease in the adsorption of Aizoon components on the SS340 surface at high temperatures. However, the Aizoon extract still shows an appreciable inhibition at 318 K equal to 85.6% with 300 ppm extract.

**Table 1:** Dissolution parameters at different temperatures for SS430/2 M HCl system in different Aizoon conc. measured from mass reduction test

Temp. K	Conc., (ppm)	MR, mg	CR, mm per y	*θ*	%*I*
298	0.0	52.7 ± 0.020	39.2 ± 0.011	—	—
	50	12.9 ± 0.015	9.6 ± 0.017	0.755	75.5
	100	8.8 ± 0.017	6.2 ± 0.011	0.840	84.0
	150	5.1 ± 0.024	3.6 ± 0.025	0.908	90.8
	200	3.1 ± 0.014	2.2 ± 0.023	0.944	94.4
	250	2.5 ± 0.031	1.8 ± 0.022	0.955	95.5
	300	2.4 ± 0.020	1.7 ± 0.018	0.958	95.8
303	0.0	97.3 ± 0.011	72.4 ± 0.019	—	—
	50	32.2 ± 0.024	22.7 ± 0.012	0.730	73.0
	100	21.2 ± 0.023	15.0 ± 0.014	0.820	82.0
	150	13.8 ± 0.018	9.7 ± 0.015	0.883	88.3
	200	9.5 ± 0.015	6.7 ± 0.012	0.920	92.0
	250	9.3 ± 0.021	6.6 ± 0.025	0.923	92.3
	300	8.3 ± 0.015	5.9 ± 0.017	0.930	93.0
308	0.0	173.1 ± 0.014	128.7 ± 0.015	—	—
	50	59.4 ± 0.012	41.9 ± 0.020	0.674	67.4
	100	33.4 ± 0.013	23.6 ± 0.022	0.816	81.6
	150	20.7 ± 0.010	14.6 ± 0.014	0.886	88.6
	200	17.2 ± 0.024	12.1 ± 0.018	0.905	90.5
	250	15.4 ± 0.022	10.9 ± 0.021	0.915	91.5
	300	13.9 ± 0.011	9.8 ± 0.022	0.923	92.3
313	0.0	235.0 ± 0.014	174.8 ± 0.014	—	—
	50	81.9 ± 0.012	57.9 ± 0.013	0.620	62.0
	100	57.1 ± 0.022	40.3 ± 0.011	0.735	73.5
	150	41.9 ± 0.025	29.6 ± 0.012	0.805	80.5
	200	29.5 ± 0.027	20.8 ± 0.010	0.863	86.3
	250	25.4 ± 0.024	17.9 ± 0.022	0.882	88.2
	300	23.3 ± 0.017	16.5 ± 0.025	0.892	89.2
318	0.0	287.7 ± 0.025	213.9 ± 0.017	—	—
	50	146.8 ± 0.021	103.7 ± 0.019	0.590	59.0
	100	105.2 ± 0.017	74.3 ± 0.014	0.711	71.1
	150	84.1 ± 0.015	59.4 ± 0.016	0.789	78.9
	200	61.6 ± 0.013	43.5 ± 0.022	0.831	83.1
	250	56.4 ± 0.011	39.8 ± 0.020	0.845	84.5
	300	52.6 ± 0.010	37.2 ± 0.017	0.856	85.6

**Table 2:** Adsorption parameters for Aizoon extract on SS430 surface

Temp. K	*K*_ads_ M^−1^	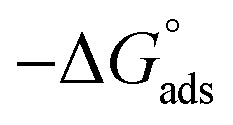 kJ mol^−1^	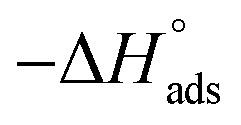 kJ mol^−1^	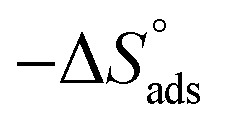 J mol^−1^K^−1^
298	86.3	20.9	47.9	90.5
303	44.8	19.6		93.3
308	40.9	19.7		91.5
313	28.2	19.1		92.1
318	18.6	18.3		93.1

### Adsorption isotherms

**Fig. 2:**
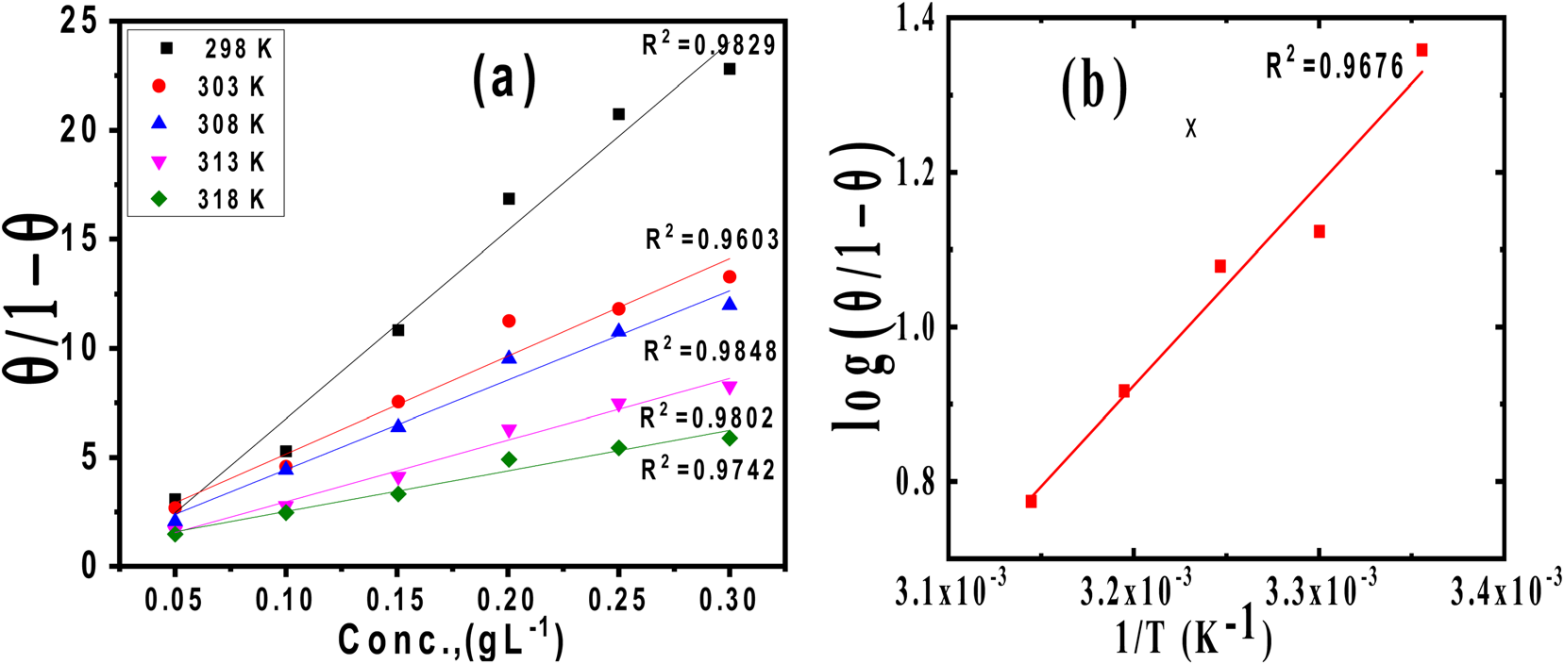
(a) Langmuir relation for adsorption of Aizoon on the surface of SS430 in 2 M HCl at different temperatures (b) the relation between log(*θ*/1 − *θ*) and (1/*T*) for SS430/2 M HCl solution with 300 ppm Aizoon extract.

The main goal of identifying the type of adsorption isotherm for the extract over a metal surface is to determine the inhibition mechanism. Different isotherm models were tested for SS430 in HCl-Aizoon mixture and it was found that the adsorption of Aizoon extract on the surface of SS430 agrees with the Langmuir type model as obeying the following equation:^26^5
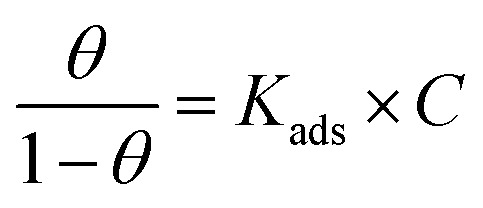
where *C* is the Aizoon concentration, and *K*_ads_ is the equilibrium constant of the adsorption process. The relation between 
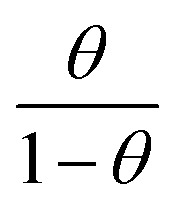
 and *C* in Fig. 2(a) gives straight lines confirming the Langmuir model for Aizoon adsorption at all studied temperatures. Therefore, a monolayer of Aizoon extract is formed on the SS430 surface and there is no interaction between the adsorbed molecules on the SS430 surface. “The standard Gibbs function of adsorption–desorption process 
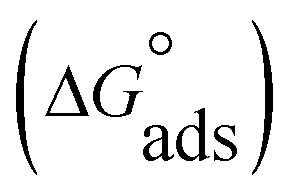
 at different temperatures (Table 2) can be calculated according to the following equation:^27^6
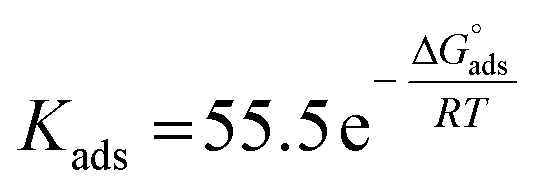
where 55.5 is the strength of water in the solution, *R* is 8.314 J K^−1^ mol^−1^ and *T* is the temperature in kelvin. It was found that the value of 
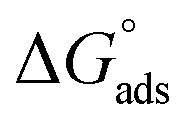
 is −20.9 kJ mol^−1^ at 298 K. The computed 
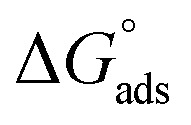
 values in the current study are virtually slightly lowered from less negative to more negative than −20 kJ mol^−1^, demonstrating that the adsorption of the extract molecules is not just physical or chemical, but also a thorough adsorption (physical and chemical adsorption). It was discovered that there was a little drop in the absolute value of 
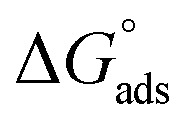
 when test temperature rose, suggesting that the adsorption was slightly unfavorable and that physical adsorption played a larger role in the adsorption process than chemical adsorption.^28^ Moreover, the increase in the equilibrium constant with temperature (Table 2) indicates that the rate of desorption increased at a high temperature which is additional evidence that the adsorption of Aizoon extracts on SS430 surface is physical in type (see Fig. 2(a)).

The enthalpy of adsorption 
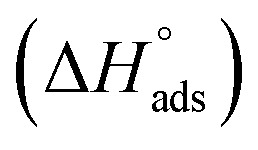
 of Aizoon extract on SS430 surface in 2 M HCl at 298 K can be calculated from eqn (7)7
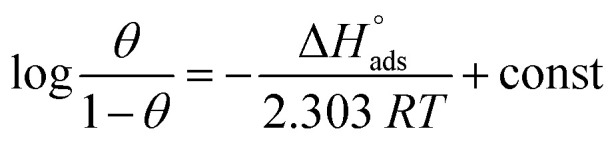


Fig. 2(b) shows the relation between 
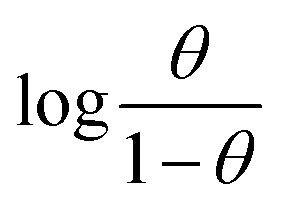
*vs.* 1/*T* for SS430 in 2 M HCl solution. The average value of 
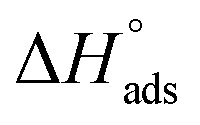
 is computed from the slope of the fitted line and is equal to −47.9 kJ mol^−1^. Therefore, extract adsorption on the SS430 surface is accompanied by a releasing heat, confirming the spontaneity of the adsorption process. The stranded entropy 
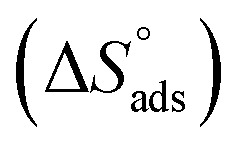
 for the adsorption process can be estimated from the following equation:8
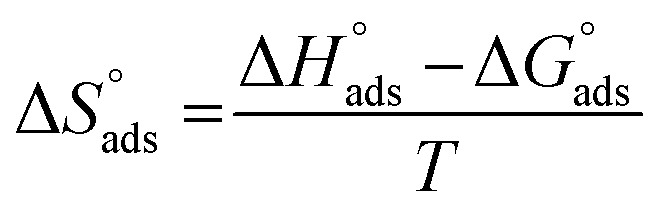


Table 2 contains the obtained 
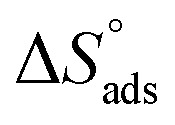
 values. 
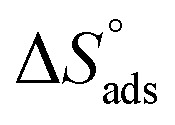
 fall from −90.5 to −93.1 J mol^−1^ K^−1^ when the temperature increases from 298 to 318 K. This finding suggests that the ordering rises with temperature due to the Aizoon extract's ability to replace a large number of water molecules.^29^

### Kinetic and thermodynamic parameters

**Fig. 3:**
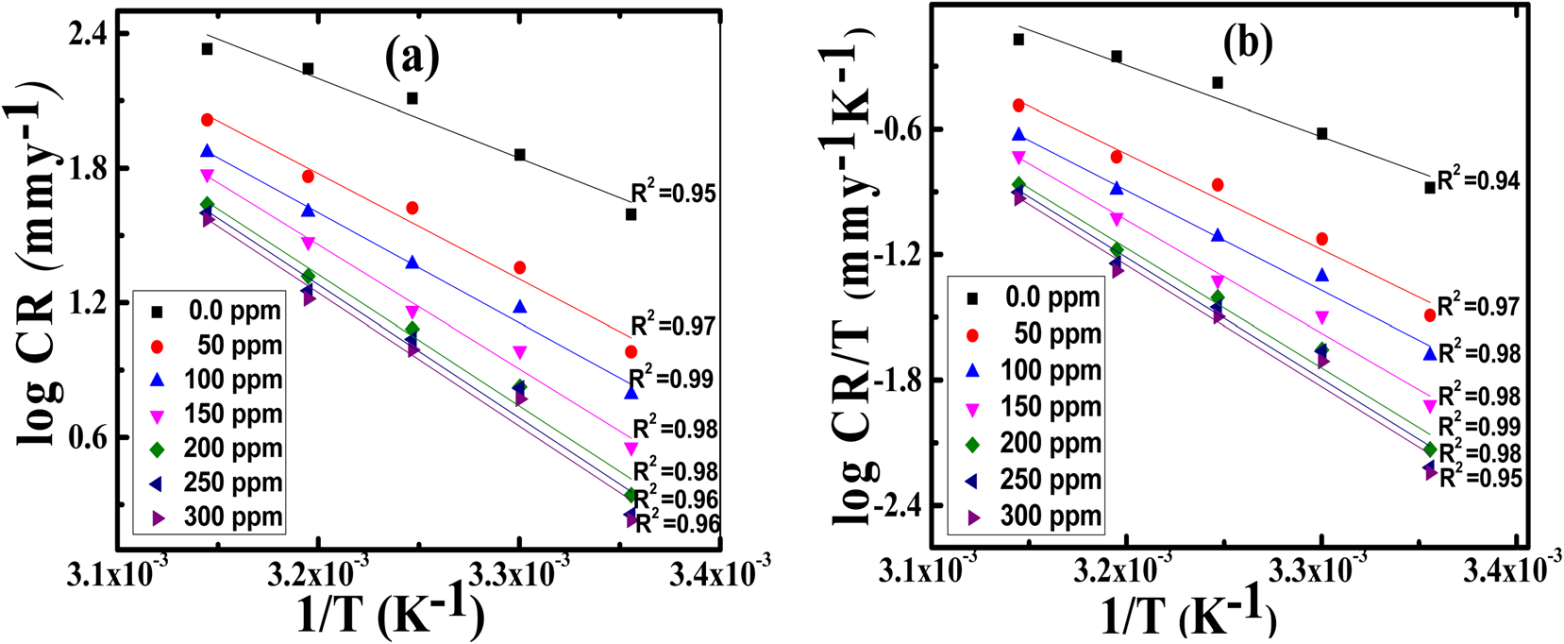
Arrhenius plots of (a) log CR and (b) log CR/*T* against 1/*T* for SS430/2 M HCl with different Aizoon concentrations.

Arrhenius equation can be used to calculate the activation energy for corrosion process according to the relation between CR and activation energy 
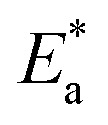
 as in eqn (9)9
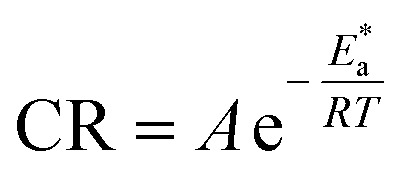
where CR in mm per y is obtained from the mass reduction test and A is the pre-exponential constant. The logarithm of CR is plotted as a function of 1/*T* in Fig. 3(a). The slope of the fitted lines gives the 
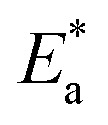
 for corrosion process of SS430/2 M HCl system at different Aizoon concentrations. The increase in 
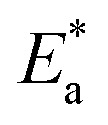
 for corrosion process with increasing Aizoon content in the corrosive medium, as shown in the Table 3 indicates that the Aizoon extract hindrance the corrosion of SS430 because of adsorption of Aizoon compounds on the SS430 surface. The thermodynamic parameter of activation ΔH* and ΔS* for the corrosion process are calculated from eqn (10).10
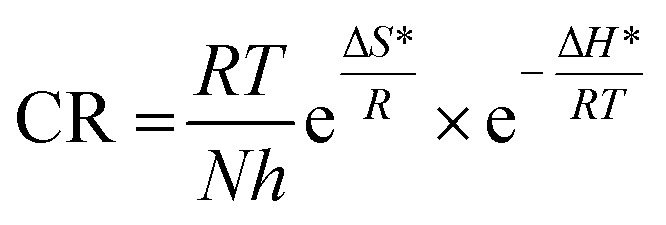
Where *h* is Planck's constant and *N* is Avogadro's number. “Fig. 3(b) is the relation between log CR/*T* and 1/*T*. ΔH* and ΔS* for SS430 corrosion in 2 M hydrochloric acid with different Aizoon concentrations are estimated from the slope and the intercept of the fitted lines in Fig. 3(b), respectively and their values are summarized in Table 3. The positive values of ΔH* both in the absence and presence of extract reflect the endothermic nature of the SS430 dissolution process and indicate that the dissolution of SS430 is difficult.^30^ However, an increment is observed in the positive value of ΔH* with increasing the extracted content in the corrosive medium indicating that the corrosion reaction needs higher energy to proceed which confirms the inhibition effect of Aizoon extract. It can be observed in Table 3 that 
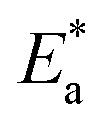
 and ΔH* vary in the same manner but the values of ΔH* are lower than the corresponding 
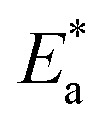
. This already-reported effect confirms that the corrosion process must involve a gaseous reaction.^31^ Furthermore, the value of ΔS* is positive and increases significantly in the corrosive medium containing the Aizoon extract compared to that in the free acid solution. This elucidates that the disorder increment occurs in transferring from reactants to the activated complex on the metal/solution interface. This is due to the replacement of solvent molecules in the medium which increase the randomness of the system.

**Table 3:** Activation factors for SS430 corrosion with and without altered concentrations of Aizoon extract in 2 M HCl

Concentration (ppm)	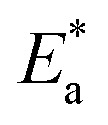 (kJ mol^−1^)	ΔH* (kJ mol^−1^)	ΔS* (J mol^−1^ K^−1^)
Blank	67.6 ± 0.2028	65.1 ± 0.2333	65.0 ± 0.2729
50	90.0 ± 0.2028	87.4 ± 0.1453	68.4 ± 0.1453
100	94.0 ± 0.1732	91.4 ± 0.2404	77.9 ± 0.1528
150	106.0 ± 0.2603	103.4 ± 0.2028	113.6 ± 0.1856
200	112.1 ± 0.1528	109.6 ± 0.2309	130.6 ± 0.1732
250	113.6 ± 0.2048	111.0 ± 0.2333	134.4 ± 0.1528
300	113.7 ± 0.2309	111.1 ± 0.1764	134.0 ± 0.1453

### Electrochemical impedance spectroscopy (EIS) tests

**Fig. 4:**
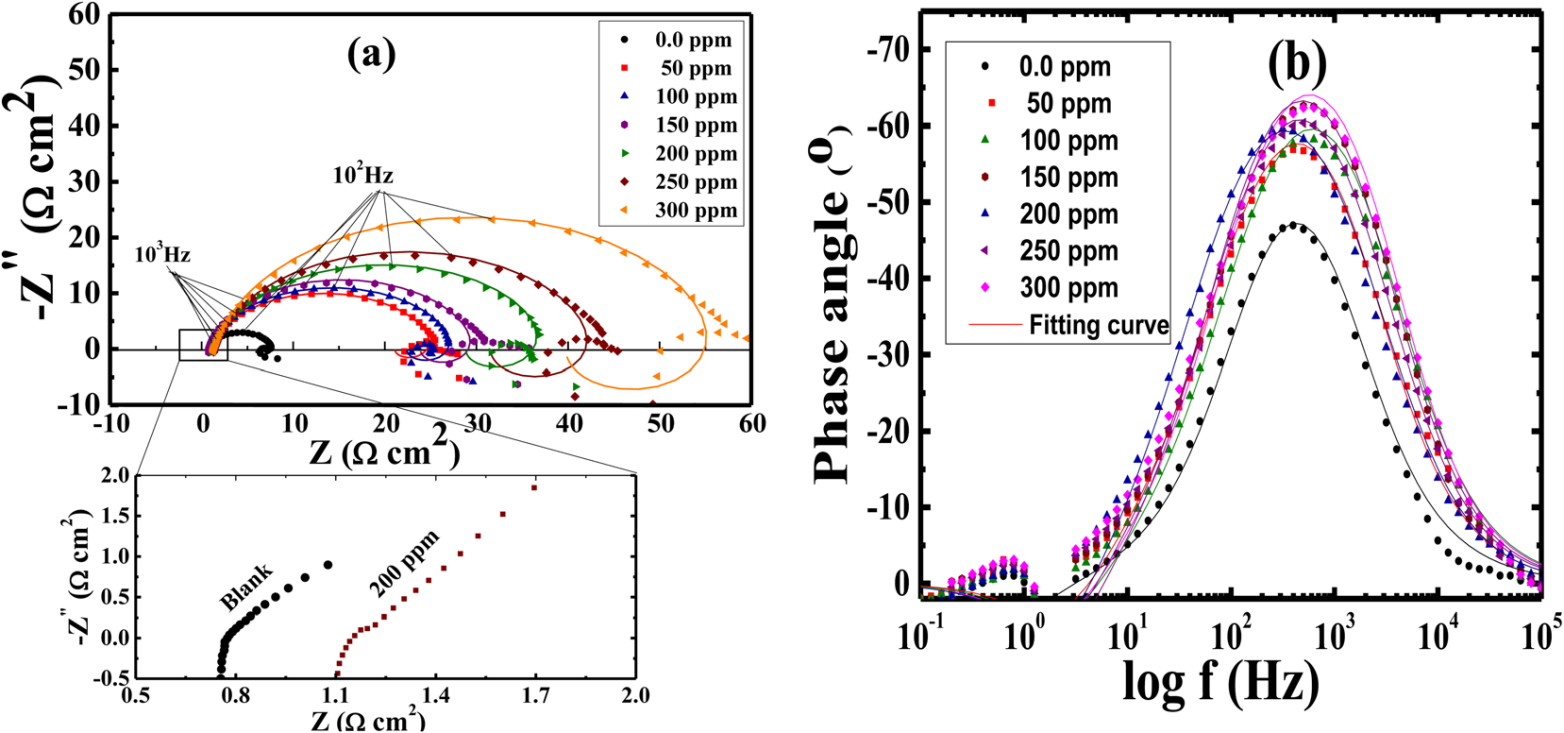
Impedance spectra (a) and phase angle (b) *vs.* log *f* for SS430 in 2 M HCl with different Aizoon concentrations at 298 K. The symbols represent the experimental data, while solid lines are the fitted curves using the modified equivalent circuit.

**Fig. 5:**
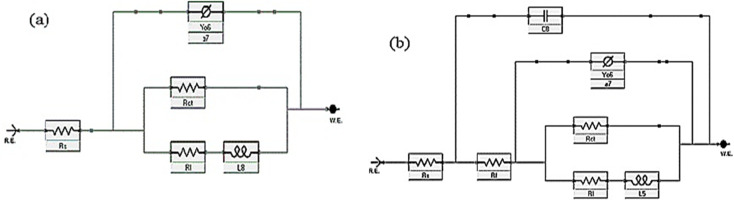
Equivalent circuit diagrams used to fit impedance data for SS430/2 M HCl (a) without and (b) with extract at 298 K.

The corrosion action of SS430 in 2 M HCl system in the presence of different Aizoon concentrations (0–300 ppm) was studied by EIS at 298 K and the spectra are depicted in Fig. 4(a and b) for Nyquist and phase angle, respectively. The impedance spectra for SS430 in 2 M HCl show two-time constant related to one charge transfer reaction and the other is for a small conductive loop at the low-frequency region. On the other hand, the spectra for the SS430/2 M HCl system in the presence of the extract exhibit an additional time constant on the high-frequency region which is related to film formation and this capacitive loop becomes more noticeable at high extract concentration and overlaps with the charge transfer loop at low concentration of extract (see Fig. 4(a)). The inductive loop at low frequency domain could be related to the relaxation process of adsorbed species as hydrogen cation, chloride anion and/or Aizoon components on the electrode surface, or to the decay of the passive layer on SS430 surface at low frequency.^32–34^ Different electrical circuits were examined to fit the data collected from EIS measurements. The two circuits in the Fig. 5(a and b) showed the best agreement with the data obtained from EIS for SS430/HCl system in the blank and acid solution containing the extract, respectively. The equivalent circuit components are resistance of the solution (*R*_s_), film resistance (*R*_f_), film capacitance (*C*_f_), constant phase element (CPE), charge transfer resistance (*R*_ct_) and resistance (*R*_L_) associated with the inductive element (L). The capacitor in the electrical circuit was replaced by (CFE) to compensate for surface heterogeneity. The capacitance double layer (*C*_dl_), and inhibition percent (*I*%) were calculated using the following eqn (11) & (12), respectively:

11 *C*_dl_ = [*Y*_0_ (*R*_ct_)^1−*n*^]^1/*n*^

where (*Y*_0_) is the proportional factor, (*R*_ct_) charge transfer resistance and *n* is an exponential term which is surface morphology dependent.12
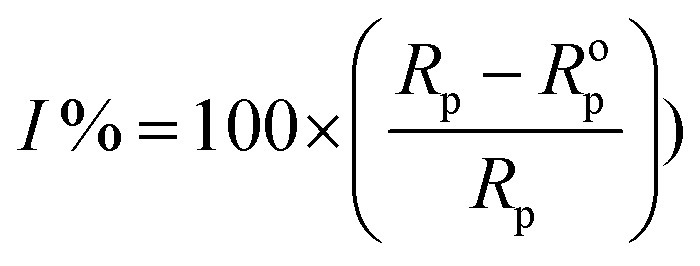
where *R*_p_ and 
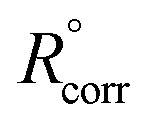
 are the polarization resistance with and without Aizoon extract, respectively.

13 *R*_p_ = *R*_ct_ + *R*_f_

14
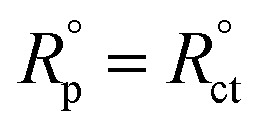
where *R*_ct_ and 
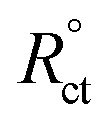
 are the charge transfer resistances with and without Aizoon extract, respectively and *R*_f_ is the film resistance. Extracted parameters are represented in Table 4. It can be observed that the polarization resistance (*R*_p_) measured from EIS for SS430/2 M HCl at different Aizoon concentrations in a great consistency with the *R*_p_ estimated from PDP measurements and reported in the next section (see Tables 4 and 5). Both the film and charge transfer resistances increase with increasing Aizoon concentration, see Table 4, demonstrating the decrease in the rate of SS430 in the presence of Aizoon extract. However, the *R*_ct_ is much higher than *R*_f_ in all Aizoon concentrations, indicating that the corrosion mechanism is under control of charge transfer and only a thin film is formed.^35^ The value of the exponential term (*n*) which reflects the surface homogeneity is decreasing significantly in the presence of Aizoon components compared to its value in the free acid confirming the film formation on SS430 surface. In other words, the surface of SS430 becomes less homogenous because of the growth of a thin film of extract on it. Moreover, the value of *n* shows a slight increase with increasing Aizoon concentration and this means that the film formation increases which leads to enhancement in the surface homogeneity. The capacitance double layer value decreases by increasing of Aizoon extract concentration because of the increase in the thickness of the electrical double layer formed on the SS430 surface and/or the decrease in dielectric constant of corrosive medium which can be explained by removal of water molecules by extract molecules according to the Helmholtz eqn (6)15
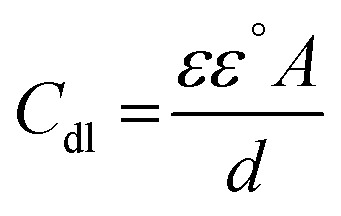
where *ε* is the corrosive medium dielectric constant, *ε*^o^ is the permittivity of the vacuum, *d* is the protective layer thickness and A is the electrode surface area”. Furthermore, the maximum phase angle at high-frequency domain moved to more negative value with rising the extract amount in the corrosive medium (see Fig. 4(b)), proving that Aizoon acts as a barrier providing a better protection for SS430 from corrosion at high extract concentration.

**Table 4:** Electrochemical parameters obtained from EIS test for the corrosion of SS430 in 2 M HCl at different concentrations of Aizoon and 298 K

Conc. ppm	*R*_s_, ohm cm^2^	*R*_f_, ohm cm^2^	*R*_ct_ ohm cm^2^	*R*_L_ ohm cm^2^	*R*_p_ = *R*_ct_ + *R*_f_ Ω cm^2^	*L* (H)	*C*_f_ *×* 10^−6^ (F)	*Y*_o_ *×* 10^−6^	*n*	*C*_dl_ (F)	*I*%
Blank	0.788 ± 0.0013	—	6.9 ± 0.0011	17.9 ± 0.0230	6.950	9.408	—	384	0.896	192.0 ± 0.145	—
50	1.031 ± 0.0011	0.630	25.0 ± 0.021	88.0 ± 0.0155	25.630	6.980	49.8	328	0.735	46.2 ± 0.173	72.9
100	0.966 ± 0.0012	0.970	26.7 ± 0.017	88.2 ± 0.0239	27.67	6.971	35.8	223	0.736	32.0 ± 0.156	74.9
150	0.810 ± 0.0015	1.351	28.8 ± 0.023	88.4 ± 0.0153	30.191	7.637	45.6	205	0.738	25.7 ± 0.120	77.0
200	1.178 ± 0.0021	1.877	36.2 ± 0.015	85.9 ± 0.0203	38.077	7.250	20.5	155	0.740	23.7 ± 0.173	81.8
250	1.400 ± 0.0020	2.191	41.5 ± 0.013	88.7 ± 0.0145	43.691	9.910	20.2	125	0.748	19.3 ± 0.120	84.1
300	1.700 ± 0.0011	3.591	52.8 ± 0.017	100 ± 0.0161	56.421	10.40	25.6	95	0.753	16.7 ± 0.230	87.7

**Table 5:** Polarization parameter obtained from Tafel curves for SS430 in 2 M HCl with different concentration of Aizoon at 298 K

Conc., ppm	−*E*_corr_ mV *vs.* SCE	*β*_a_ mV dec^−1^	−*β*_c_ mV dec^−1^	*i*_corr_ μA cm^−2^	CR mm per y	%*I*	*R*_p_ ohm cm^2^	(1 − *α*)	*i*_o_ μA cm^−2^
Blank	528 ± 0.141	91 ± 0.272	126 ± 0.142	3980 ± 0.202	45.08	—	5.77 ± 0.015	0.468	3370 ± 0.241
50	498 ± 0.260	67 ± 0.172	122 ± 0.182	660 ± 0.278	7.48	83.4	28.32 ± 0.022	0.486	627 ± 0.182
100	501 ± 0.175	58 ± 0.252	118 ± 0.172	621 ± 0.241	7.03	84.4	27.24 ± 0.014	0.503	570 ± 0.172
150	482 ± 0.115	63 ± 0.122	120 ± 0.208	575 ± 0.241	6.51	85.6	31.02 ± 0.024	0.501	511 ± 0.205
200	487 ± 0.188	66 ± 0.252	120 ± 0.202	511 ± 0.115	5.79	87.2	36.04 ± 0.013	0.494	460 ± 0.115
250	514 ± 0.240	73 ± 0.147	119 ± 0.272	409 ± 0.157	4.63	89.7	47.93 ± 0.022	0.497	393 ± 0.241
300	510 ± 0.218	71 ± 0.278	121 ± 0.282	280 ± 0.241	3.17	93.0	69.55 ± 0.016	0.490	246 ± 0.278

### Potentiodynamic polarization (PDP) tests

**Fig. 6:**
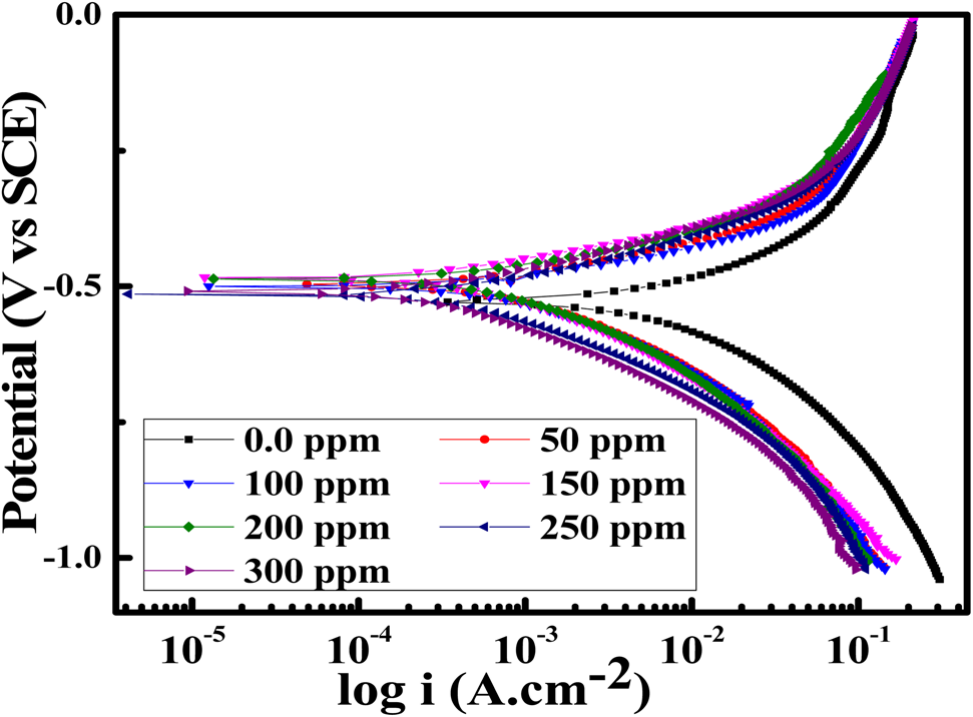
PDP curves for the corrosion of SS430 in 2 M HCl with different concentrations of Aizoon extract at 298 K.

PDP measurements were done for the SS430 electrode in 2 M HCl to evaluate the electrochemical kinetic behavior with different concentrations from Aizoon extract. Fig. 6 shows the Tafel curves for the SS430/HCl system at different concentrations of Aizoon. The corrosion potential (*E*_corr_) and corrosion current density (*i*_corr_) are estimated from the coordinates of the point when the linear part of the anodic and cathodic curves intersected. All the electrokinetic parameters obtained from Tafel extrapolation are summarized in Table 5. It can be noticed that the cathodic curves in the Tafel plot for SS430 are parallel in both the extract-free acid and in different concentrations of extract indicating that the addition of Aizoon extract does not alter the mechanism of the hydrogen reduction reaction.^36^ On the other hand, the anodic Tafel slope decreases significantly in the extract-acid mixture compared to that in the blank corrosive medium, confirming the change in the metal dissolution mechanism by the addition of the extract (see Fig. 6 and Table 5). However, the addition of extract leads to a considerable shift in the *i*_corr_ to a lower value compared with that in free acid and the observed trend is concentration-dependent. Moreover, the value of *E*_corr_ for the SS430/HCl system changes to lower negative values with Aizoon extract because the SS430 in extract-free acid starts to be corroded first. In other words, the presence of the extract retards the SS430 corrosion in 2 M hydrochloric acid solution (see Table 5). The inhibition percent (*I*%) and the surface coverage (*θ*) in different extract concentrations have been calculated using the following equations.16
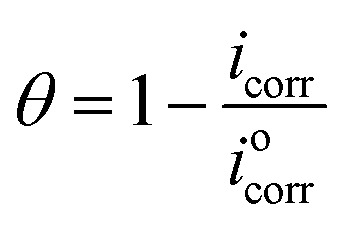

17 *I*% = *θ* × 100

**Fig. 7:**
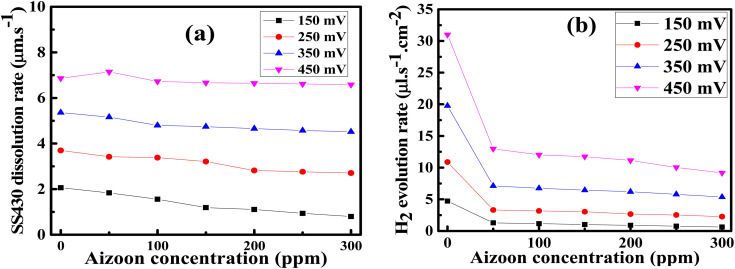
Rates of SS430 dissolution (a) and hydrogen evolution (b) under different anodic and cathodic overpotential, respectively for SS340/2 M HCl system as a function of extract concentration at 298 K.

“where, 
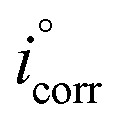
 and *i*_corr_ are the corrosion current densities without and with Aizoon extract, respectively”. The %*I* increases, and the CR decreases with increasing the extract concentration and this result is comparable with that obtained from MR tests at 298 K (see Tables 2 and 5). The values of current at equilibrium 
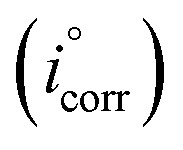
 are comparable to the values of corrosion current densities (*i*_corr_) (Table 5). This illustrates that the constituent components of Aizoon extract are successfully adsorbed on the SS430 surface which in turn close the active sites and retard the rate of corrosion. Furthermore, the polarization resistances (*R*_p_) for SS430/HCl system with different concentrations of Aizoon were calculated according to the following equation:18
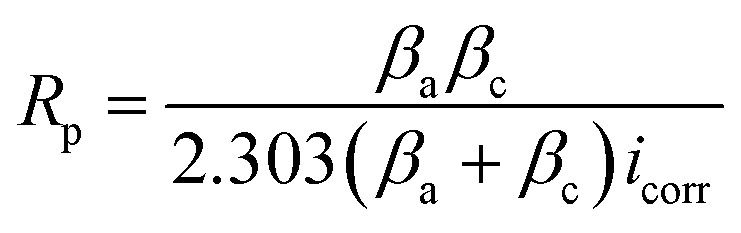
where *i*_corr_ is the corrosion current density and *β*_a_, *β*_c_ are the anodic and cathodic Tafel slope, respectively. The *R*_p_ increases significantly with increasing extract concentration, confirming the formation of the adsorbed layer of Aizoon extract on the SS430 surface which increases with increasing extract concentration, as it can be seen from the surface coverage (*θ*), hindering the rate of corrosion process for SS430 (see Table 5). The rate of SS430 dissolution and hydrogen evolution under different anodic and cathodic overpotential for SS430/2 M HCl system in different extract concentrations were obtained from Faraday's equation and then depicted as a function of extract concentration at 298 K in Fig. 7(a) and (b), respectively. The rate of metal oxidation and hydrogen reduction increase as expected with increasing the applied anodic or cathodic overpotential, respectively. The addition of extract leads to lower rate of anodic and cathodic reaction at overpotential below 400 mV. This demonstrates that the Aizoon extract is adsorbed on anodic and cathodic sites on SS430 surface. Otherwise, at anodic overpotential above 400 mV there is no observable change in SS430 dissolution rate by addition of Aizoon extract (see Fig. 7(a)). This means that the extract molecules were desorbed from anodic sites at that high anodic overpotential, resulting in no shift in the current density with the addition of different extract concentrations. However, above 400 mV cathodic overpotential there was a considerable decrease in the hydrogen evolution rate with the addition of Aizoon extract to SS430/2 M HCl system (see Fig. 7(b)) which indicates that the components of Aizoon extract are strongly adsorbed to cathodic sites even at high overpotential. Moreover, the decrease in the rate of hydrogen evolution is higher with the addition of Aizoon extract compared to the decrease in SS430 dissolution rate as observed in Fig. 7(a) and (b). Since the value of *E*_corr_ with and without adding extract is less than 85 mV (46 mV), the extract acts as a mixed type inhibitor tending to be superior for inhibiting the cathodic reaction (*β*_c_ > *β*_a_).

### Effect of extract on cathodic reaction

The cathodic reaction for metal corrosion in an acidic medium is mainly a hydrogen evolution reaction (HER). The HER in acidic solution proceeds in three steps proton reduction (Volmer reaction), electrochemical desorption (Heyrovsky reaction) and/or chemical desorption (Tafel reaction) as represented by the following equations.*H*^+^ + e^−^ ⇌ *H*_ads_ (Volmer reaction)*H*^+^ + *H*_ads_ + e^−^ ⇌ *H*_2_ (Heyrovsky reaction)*H*_ads_ + *H*_ads_ ⇌ *H*_2_ (Tafel reaction)

Thus, the whole reaction takes place through the integration of two probable reaction steps, Volmer–Heyrovsky and Volmer–Tafel. “The Tafel slope is a good approximation for defining the rate-determining step for (HER). The kinetic models for HER assumes that the rate-determining step is Volmer, Heyrovsky or Tafel reaction if the Tafel slope is about 120, 40 or 30 mV dec^−1^, respectively.^37^ The estimated cathodic Tafel slopes in this study result in the range 126.4–117.5 mV dec^−1^ demonstrating that the rate-determining step for HER over SS430 electrode in 2 M HCl with or without Aizoon extract is Volmer step. The exchange current density (*i*_o_) and charge–transfer coefficient (1 − *∝*) for HER for the examined systems have been calculated from Tafel equations.

19 *η* = *a* + *b* log *i*

20
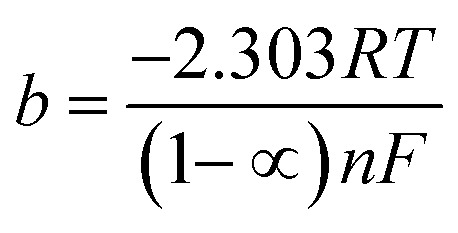
21
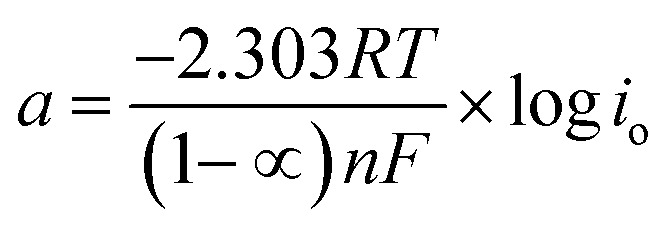
where *η i*s the applied overpotential, *i* is the current density, *b* is the Tafel slope, *a* is the intercept. *R* is 8.314 J mol^−1^ K^−1^, *∝* is the symmetry factor, *n* is the number of electrons exchanged and *F* is 96 485 Coulomb mol^−1^. As listed in Table 5 the value of charge-transfer coefficients for all cases is approaching to 0.5 which gives additional evidence that the Volmer step is the rate-determining step.^38^ The exchange current density for HER over SS430 in 2 M HCl decreases significantly by raising the concentration of Aizoon extract, indicating that the rate of HER decreases in the existence of Aizoon extract. Accordingly, as the Volmer step is the rate-determining step for hydrogen evolution reaction, the active cathodic sites for hydrogen adsorption on SS430 electrode decrease because of extract adsorption on these sites. In another word, the presence of extract decreases the surface area for hydrogen adsorption and slows down the charge transfer reaction.

### Scanning electron microscope (SEM) analysis

**Fig. 8:**
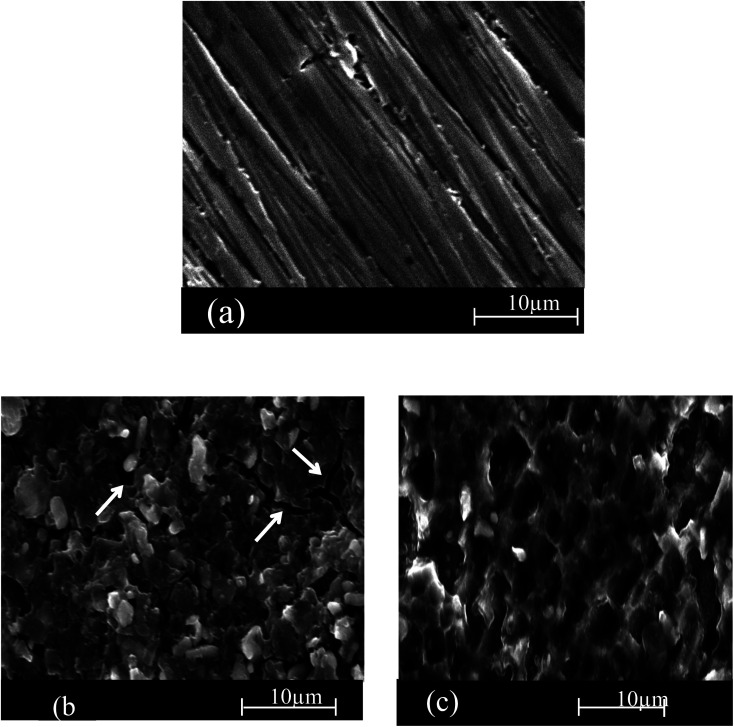
SEM micrographs of SS 430 surface before of dipping in 2 M HCl (a) and after 3 h of dipping in (b) 2 M HCl and (b) 2 M HCl + 300 ppm of Aizoon extract (c) at 298 K.

Scanning electron was conducted to elaborate a connection between the experimental data and the morphological shape of the SS430 surface. Fig. 8(a–c) are the SEM images for the as-polished SS430 surface and the surface after 3 h immersion in acid without extract and in acid containing 300 ppm of Aizoon extract at 298 K, respectively. The images in Fig. 8(b) show a noticeable crack on the alloy surface because of sever attack of the corrosive medium when not protected by the extract. On the other hand, in the acid solution containing the extract, the cracks are not observed and the surface of SS430 becomes smoother compared to the surface of SS430 in hydrochloric acid without extract (see Fig. 8(c)). This demonstrates that the Aizoon extract retards the corrosion of SS430 through the adsorption of its components on the SS430 surface.

### Fourier transforms infrared (FTIR) analysis

**Fig. 9:**
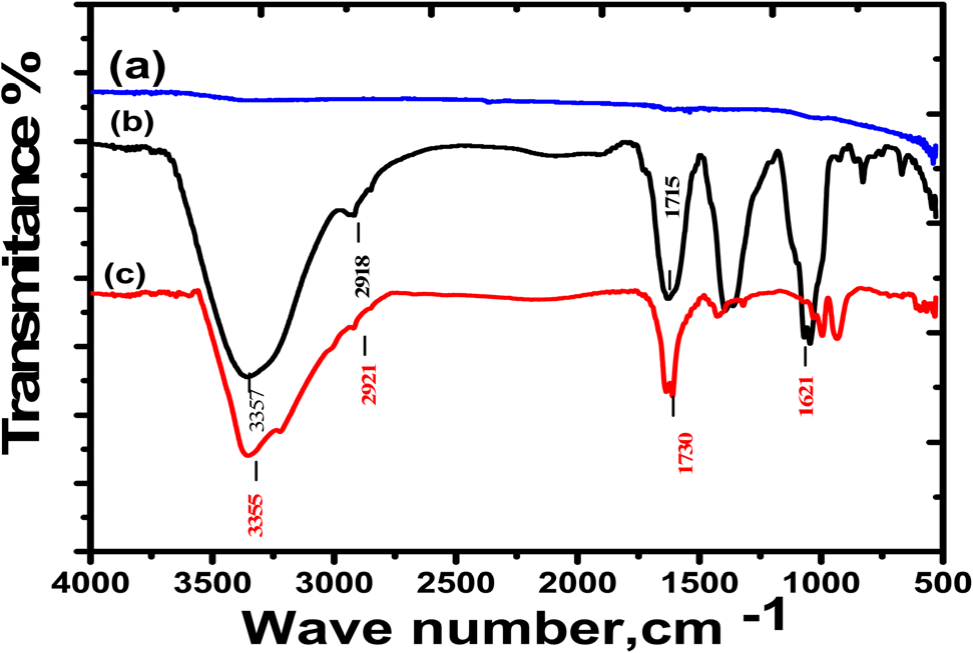
FTIR spectra for (a) as-pretreated SS430 surface, (b) Aizoon extract, and (c) SS430 surface after submersion in 2 M HCl + 300 ppm Aizoon for 3 h at 298 K.

**Fig. 10:**
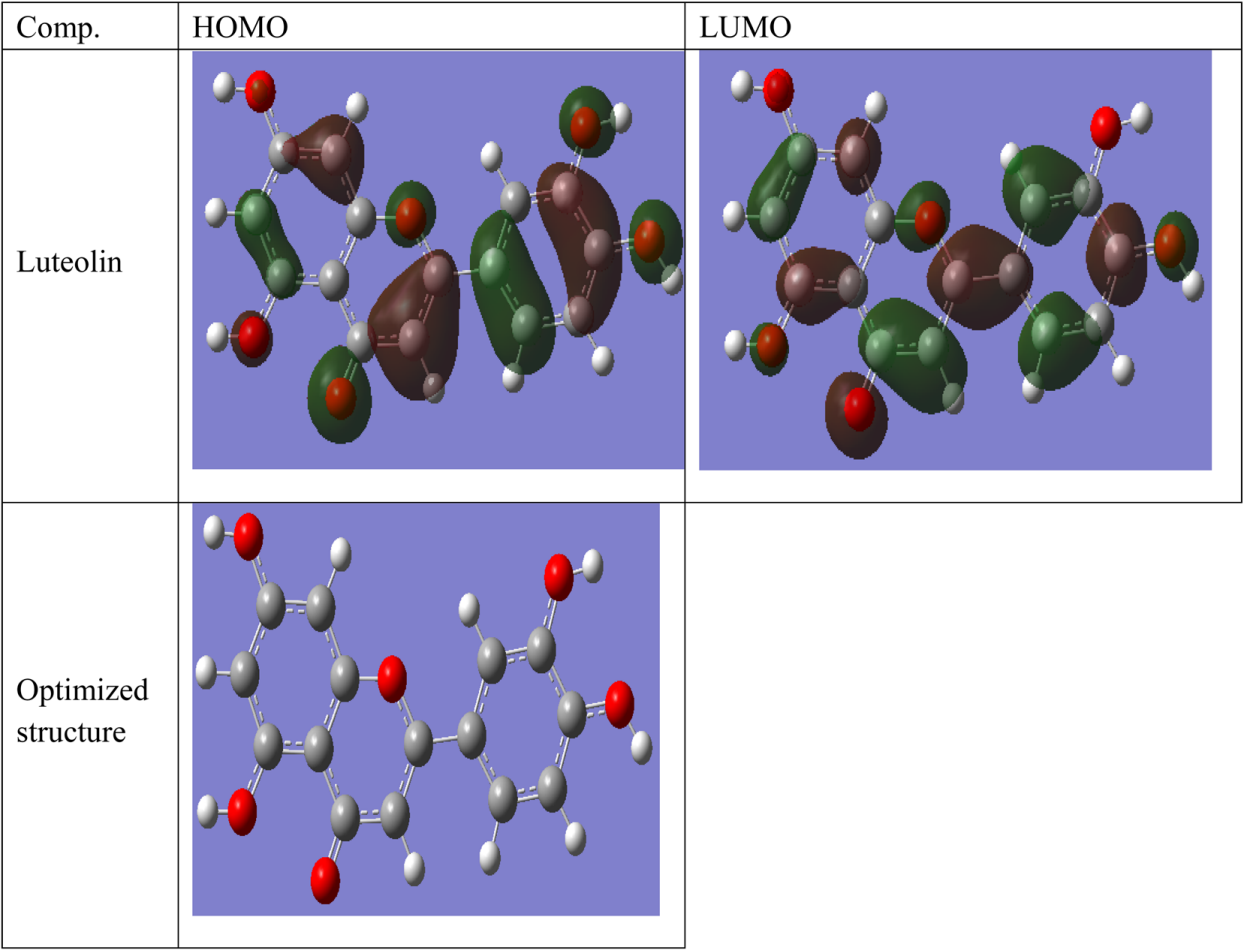
Frontier molecular orbital and electrostatic potential surface distribution for loaded form of Luteolin.

FTIR measurements were conducted to obtain a clear evaluation for the possibility of the extract forming a thin film over the SS430 surface. FTIR spectra for the Aizoon extract and SS430 surface before and after submersion in a mixture of 2 M HCl + 300 ppm extract for 3 hours at 298 K are presented in Fig. 9. It can be observed that there are no peaks appeared when the SS430 surface was examined before immersion in the mixture as in Fig. 10(a). However, the SS430 sample that was immersed in the mixture shows IR peaks like those for pure Aizoon extract with a noticeable change in both intensity and wave number (see Fig. 9(b) and (c)). The broad band at 3355 cm^−1^, related to O–H stretching, is shifted to 3371 cm^−1^ after the submersion which means that the O–H bond strength increase, and its bond length decreases due to the presence of adsorbed O atoms over the SS430 surface.^39^ Also, the peak related to C–H shifts from 2918 to 2921 cm^−1^. Moreover, the peak that belongs to the carbonyl group for pure Aizoon extract at 1715 cm^−1^and shifts to 1730 cm^−1^ when examined over SS430 surface after immersion in HCl-Aizoon mixture accompanied by a decrease in its intensity which supports the possibility of adsorption of Aizoon compounds on SS430 surface.^40,41^ In other words, the observed shifts in peaks can be assigned to the interaction between the extract molecules and the active sites on a metal surface. This means that the compounds of Aizoon are adsorbed successfully on the SS430 surface confirming the mixture formation of a protective thin film as discussed in the EIS section.

### Theoretical study results

Luteolin is considered the main components in Aizoon extract and consequently was selected to carry out the theoretical study.
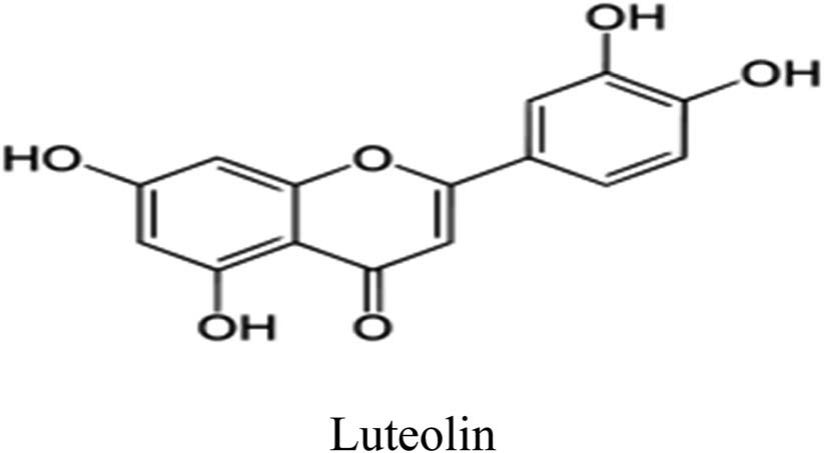


Some quantum chemical parameters which have significant impact directly on electronic interaction between the metal surface and Luteolin molecule are reported in Table 6. Fig. 10 represents frontier molecular orbital and electrostatic potential surface distribution for loaded form of Luteolin molecule. Higher *E*_HOMO_ (highest occupied molecular orbital) of the absorbent leads to higher electron donating ability. “Thus, high values of E_HOMO_ facilitate adsorption, and hence the %*I* is improved. The energy of the lowest unoccupied molecular orbital (*E*_LUMO_) indicates the ability of the molecules to accept electrons. Lowering values of E_LUMO_ the molecule would accept electrons with higher probability. The energy gap value (Δ*E*) determines the chemical reactivity. In terms of reactivity the molecule with the greater %*I* is the more active toward the SS430 surface, while the one with lower Δ*E* (Δ*E* = *E*_HOMO_ − *E*_LUMO_) is perhaps the most stable. As a result, Luteolin molecule and the Fe substrate form a stable combination. The dipole moment (*μ*) is a measure of the covalent bond polarity of the Luteolin molecule. The low dipole moment (*μ*) indicates that inhibitor molecules gather on the SS430 surface, increasing the adsorption capacity.^42^ It is agreed that the large values of dipole moment enhance the adsorption propensity of the studied compound on metal surface. The determined quantum chemical indicators of Luteolin are shown in Table 6.

**Table 6:** The determined quantum chemical indicators

Comp.	*E*_HOMO_ (eV)	*E*_LUMO_ (eV)	Δ*E* (eV)	Dipole moment (*μ*) (Debye)
Luteolin	−5.445	−1.314	4.131	2.9624

### Monte Carlo (MC) simulation

**Fig. 11:**
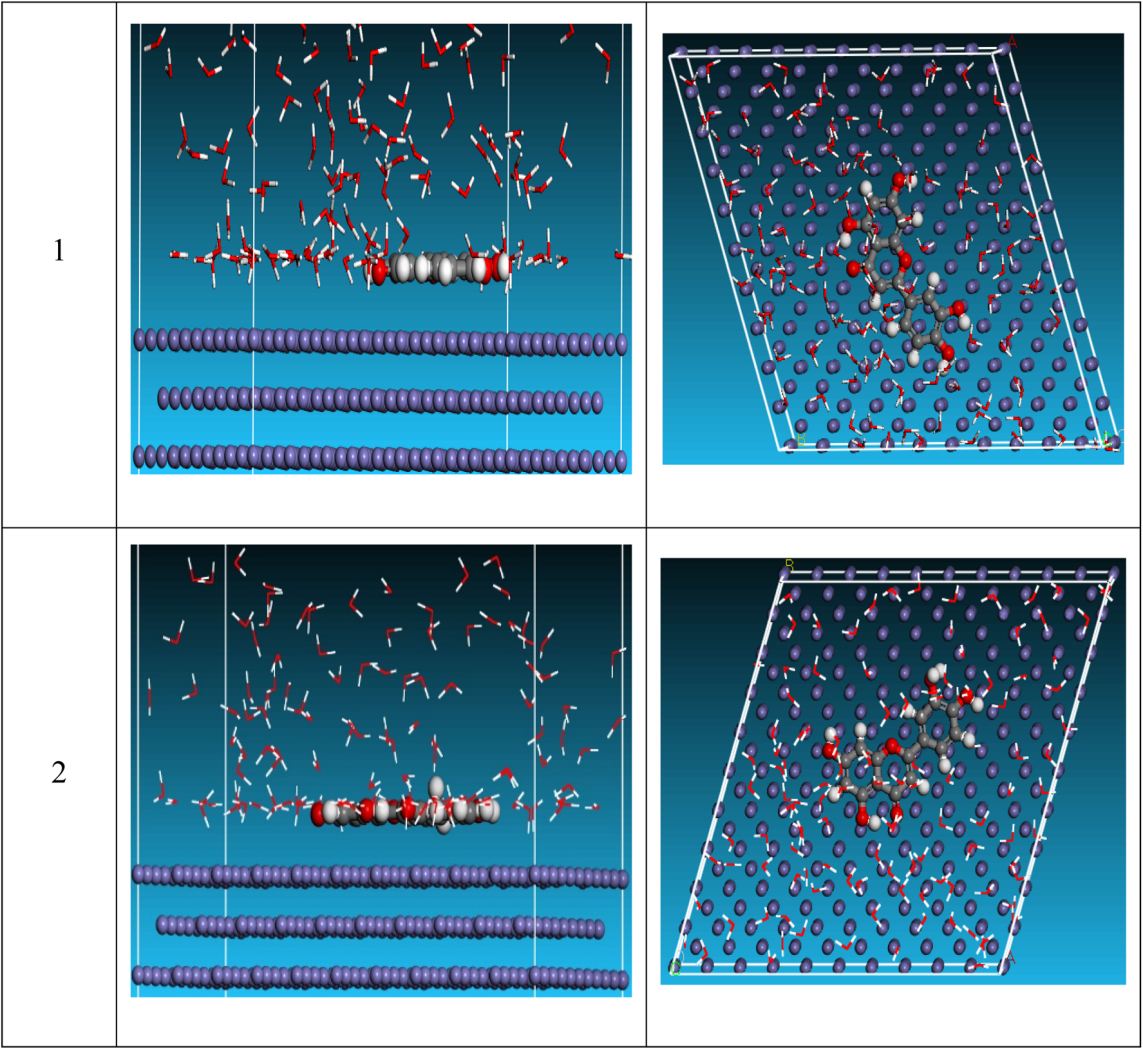
Side and top views for MD simulations with the most stable configuration of investigated compound on Fe (110) surface in vacuum conditions.

MC simulation was utilized to analyze the interaction between the examined compound and the reinforced steel surface, providing a clear image of the adsorption mechanism. “As a result, Fig. 11 depicts the side and top views of the most suitable adsorption configurations for the investigated substance protonated and unprotonated on the surface of steel, as determined by the adsorption locator module”. In addition, the total energy of the “substrate/adsorbate configuration, the adsorption energy for relaxed adsorbate molecules, rigid adsorption energy for unrelaxed adsorbate molecules, and deformation energy for relaxed adsorbate molecules were summarized in Table 7. Furthermore, the adsorption energy for the adsorbate molecules is the sum of stiff adsorption energy and deformation energy”. Table 7 demonstrates that the unprotonated form has greater adsorption energy than protonated, implying that unprotonated will have a strong adsorption on the reinforced steel surface, generating stable adsorbed layers and protecting the steel against dissolution. The d*E*_ads_/dNi value represents the energy of a metal-adsorbate arrangement with one adsorbate removed. An unprotonated molecule has a higher d*E*_ads_/dNi ratio (−206.1 kcal mol^−1^) than protonated molecule (−174.1 kcal mol^−1^)”, indicating that unprotonated molecule has more adsorption than protonated molecule. Furthermore, when compared to unprotonated and protonated water, the d*E*_ads_/dNi value for water is modest, indicating that the investigated inhibitor molecules have strong adsorption with respect to water molecules. As a result, both experimental and theoretical analyses show that unprotonated and protonated molecules are adsorbed on the reinforced surface of steel and form stable adsorbed layers, resulting in corrosion protection for the surface of the steel in the destructive environment. The order of inhibition for this inhibitor, according to MC data, is: unprotonated > protonated.

**Table 7:** MD calculated parameters most stable configuration of investigated compound on Fe (110) surface in vacuum conditions

Structures	Total energy	Adsorption energy	Rigid adsorption energy	Deformation energy	Compound d*E*_ads_/dNi	H_2_O d*E*_ads_/dNi
Fe (1 1 0) unprotonated/H_2_O	−3277.6	−3197.2	−3353.5	156.2	−206.1	−6.5
Fe (1 1 0) protonated/H_2_O	−3243.2	−3171.2	−3324.4	153.1	−174.1	−1.4

### Corrosion mechanism of Aizoon extract

**Fig. 12:**
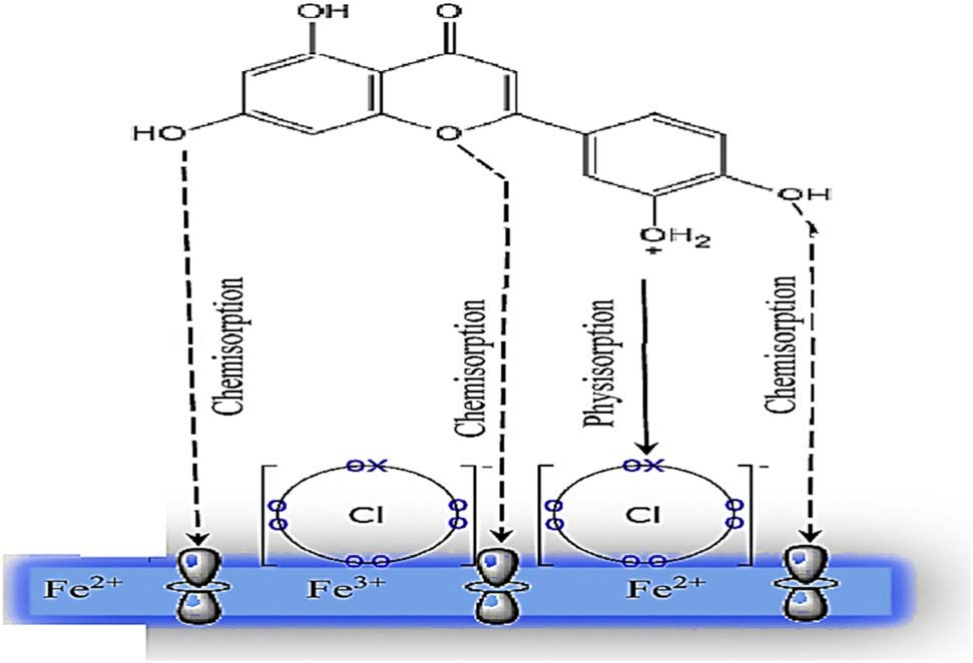
The schematic illustration of different modes of adsorption by luteolin on SS430 in 2 M HCl interface.

The role of Aizoon extract on the SS430 corrosion mitigation in HCl solution is schematically presented in Fig. 12. On the basis of molecular adsorption, the corrosion prevention of SSE430 in 2 M HCl solution by the Aizoon extract may be explained. The development of donor–acceptor surface complexes between the vacant d-orbitals of the metal and the lone pair/−electrons of the inhibitor molecules was proposed in the majority of the inhibition investigations extract molecules can be adsorbed on SS430 surface by the following ways: (i) electrostatic interaction between the charged molecules and charged metal (ii) interaction of electrons with the metal (iii) interaction of unshared pair of electrons in the molecule with the metal (iv) the combination of all previous effects.^43^ From the analysis of zero charge potential which was reported in ref. ^44^, it was indicated that the surface of SS430 carry a +ve charge in HCl solution without and with the addition of inhibitor.^45^ As previously mentioned, the investigated extract contains significant phytochemical components (*p*-hydroxybenzoic acid (**1**), gallic acid (**2**), protocatechuic acid (**3**), vallinic acid (**4**), thymine (**5**), caffeic acid (**6**), 5,7-dihydroxy chromone (**7**), pyrogallol (**8**), quercetin (**9**), kaempferol (**10**), and luteolin (**11**)) that behave as Lewis bases and form coordinate bonds with the empty d-orbitals of metal. As a result, they are adsorbed onto the metallic surface, resulting in the formation of a protective coating that shields the metal from corrosion.^46^ Hence, it has been suggested that Cl^−^ ions get adsorbed first so that an excess of negative charges is generated towards SS430 surface in corrosive environment. In the next step, the protonated Aizoon extract molecules adsorb on the metal substrate through electrostatic adsorption mechanism. The adsorbed Aizoon molecules then create a protective film which prevents the metal from coming in contact with the corrosive environment (physical adsorption).^47^ As shown in Fig. 12, the Aizoon extract molecules like 5,7-dihydroxy chromone, pyrogallol, kaempferol, protocatechuic acid,vanillic acid, thymine, caffeic acid, quercetin, *p*-hydroxy benzoic acid, gallic acid, and luteolin include plenty number of oxygen heteroatoms. These heteroatoms could donate their free electrons to the unfilled d-orbital of the iron atoms (see Fig. 12), resulting in the Aizoon extract molecules adsorption on the SS430 surface through chemisorption mechanism.^48^ The Aizoon extract's significant potential as an efficient corrosion inhibitor for SS430 in 2 M HCl is revealed by the given inhibition efficacy >95%, which is significantly higher than in comparison with some of the recent green corrosion inhibitors.

## Conclusion

Aizoon extract proved to be a good green inhibitor in corrosion mitigation of SS430 in 2 M HCl. The %*I* reaches 95.8% when the dosage is 300 ppm at 25 °C, as obtained from MR test. The corrosion inhibition is probably due to the adsorption of the extract on the metal surface without changing the mechanism of partial corrosion reaction but blocking its active sites and thus slowing down the corrosion rate. From all experiments it was found that %*I* increases with increasing Aizoon extract concentration and decreases by raising the temperature. The data obtained from this study fit well with the Langmuir adsorption isotherm. Adsorption mechanism of the Aizoon extract on the SS430 surface in 2 M HCl result combination of physical and chemical adsorptions. PDP measurement suggested that Aizoon extract can be adsorbed on both anodic and cathodic sites, but the extract preferably tends to be adsorbed on cathodic sites. Quantum chemistry (QC) computations have revealed a lot for luteolin (main component of Aizoon extract) active adsorption sites. The results of chemical and electrochemical testing are strongly supported by SEM and FTIR morphological analyses. According to molecular dynamics (MC) simulations, Aizoon adsorbs onto the Fe (1 1 0) surface through a combined physical and chemisorption mechanism, resulting in high coverage area which prevents the metal from coming in contact with the corrosive environment. This study will assist in extending the life of SS430 pipe lines, reducing corrosion costs, and reducing environmental issues. This research can be applied in the petroleum industries, as this extract protects the metals from which the petroleum utensils are made from corrosion. This leads to reduced losses in petroleum industries and industrial costs, which leads to economic growth that benefits society. The studied extract can be used safely as corrosion inhibitors for different types of steels and the study may extend to use it as corrosion inhibitor for other technical metals.

## Author contributions

Abd El-Aziz S. Fouda: supervision, conceptualization, investigation, software, validation, review and editing article, Ameena M. Al-Bonayan: methodology and taking experiment part of inhibitor and tested, Ahmed F. Molouk, writing – original draft preparation, supervision, software, resources, FMO Computations, M. Eissa: supervision, resources.

## Conflicts of interest

All Authors declare that there is not any conflict of interest in this study.

## Supplementary Material
